# Comprehensive mandatory policies are needed to fully protect all children from unhealthy food marketing

**DOI:** 10.1371/journal.pmed.1004291

**Published:** 2023-09-25

**Authors:** Francesca R. Dillman Carpentier, Fernanda Mediano Stoltze, Barry M. Popkin

**Affiliations:** 1 Hussman School of Journalism and Media, University of North Carolina, Chapel Hill, North Carolina, United States of America; 2 School of Psychology, Pontificia Universidad Catolica de Chile, Santiago, Chile; 3 Department of Nutrition, Gillings School of Global Public Health, University of North Carolina, Chapel Hill, North Carolina, United States of America

## Abstract

The World Health Organization (WHO) have released a new guideline, “*Policies to protect children from the harmful impact of food marketing”* [1] which recommends the development of comprehensive laws to reduce children’s exposure to unhealthy food marketing. This new guideline extends previous recommendations [2] to limit the adverse effects of unhealthy food marketing on the health of the world’s children. We consider here whether these new recommendations go far enough.

## Abstract 2

In this Perspective, Francesca R Dillman Carpentier and colleagues discuss the need for comprehensive policies to protect children from unhealthy food marketing and its influence on their health trajectories.

Nutrition-related noncommunicable diseases (NCDs) represent a truly global concern. Countries are increasingly responding to calls by the WHO and others to enact policies that improve diets through the reduction of foods high in added fats, sugars, and/or sodium (HFSS) [2,3]. Efforts to reduce unhealthy food marketing is included in these calls since children are disproportionately exposed to food marketing for HFSS products, which attracts the children’s attention and, ultimately, shapes their food preferences and increases their caloric intake [4,5]. The resulting dietary behaviors constitute a critical risk factor for childhood obesity [6], which can persist into adulthood and lead to cardiometabolic diseases, cancers, and other health complications [7–9].

Building on their previous recommendations published in 2010 [2], the WHO has published an updated guideline that supports food marketing policy development with considerations for implementation, monitoring, and enforcement. The guideline is based on reviews of research on children’s responses to food marketing and on the effectiveness of existing efforts to reduce unhealthy food marketing [1]. This new guideline recommends the restriction of HFSS marketing to which children may be exposed using a comprehensive definition of marketing as “any form of commercial communication, message or action that acts to advertise or otherwise promote a product or service, or its related brand” [1]. The guideline further calls for policies that are “mandatory,” “protect children of all ages” up to 18 years old, “use a government-led nutrient profile model to classify foods” for restriction, are “sufficiently comprehensive to minimize the risk of migration of marketing to other media, to other spaces within the same medium, or to other age groups,” and that “restrict the power of food marketing to persuade” [1].

The WHO makes several important advancements on its 2010 recommendations [2] in this new guideline. For one, recommendations sit on a strong justification for protecting children based on their rights to health, nutritious food, privacy, and freedom from exploitation as articulated in the United Nations’ Convention on the Rights of the Child [10]. Recommended protections are extended to children up to 18 years of age, consistent with the Convention [10], which is an important development given evidence that food marketing negatively influences both children and adolescents [4,5].

The new guideline also warns of the ineffectiveness of policies that limit food marketing restrictions to only certain types of messages (e.g., using explicit child-directed content like cartoon characters) or places of promotion (e.g., television advertising around programs made for children) [1]. The WHO’s broadened marketing definition that covers all messages to which children are exposed guards against the possible circumvention of restrictions to reach children through unrestricted avenues. The new guideline [1] also includes the important consideration of brand marketing—a marketing strategy in which a brand associated with one or more HFSS products is promoted without showing a specific product, nonetheless keeping the associated products salient in the minds of children.

Yet, the new guideline [1] could go further in its argument for comprehensiveness. For example, exposure is defined as frequency (number of times the average child is exposed to a promotion) and reach (number of children exposed to any promotion) with language focused on limiting rather than eliminating exposure. We argue that even minimal amounts of exposure risks disproportionately affecting children from lower socioeconomic strata, given documented exposure patterns [11] including a recent assessment of children’s exposure in Colombia [12]. The guideline [1] also does not fully discuss increases in health marketing, greenwashing, and social responsibility branding. This is a missed opportunity, as research shows that both children and adults may respond to marketing claims about health benefits, nutritional content, and environmental consciousness, such that they may overestimate product healthiness and find the product appealing [13,14]. This suggests a need for countries to oversee how the healthiness of foods is marketed, in addition to restricting the marketing of unhealthy foods.

This guideline [1] further misses the opportunity to support its recommendations using the wealth of research on children’s responses to marketing and the effectiveness of marketing policy. The argument for developing strong, comprehensive legislation is diluted by merging evidence from industry-led voluntary measures with evidence from mandatory regulations, such as those in Chile. Support for food marketing restrictions is therefore tentative, with reminders throughout that voluntary efforts are largely ineffective compared to mandatory policies. Recommendations also rely on a systematic review framework (GRADE) that favors randomized controlled trials (RCTs) (the gold standard for determining intervention effects) over observational research—the prevailing research design for assessing real-world outcomes. The prioritization of RCTs over real-world evidence results in the labeling of recommendations as conditional. Yet, as noted in the precautionary principle [15], postponement of interventions is not advisable if available evidence suggests a benefit to children or to public health.

The evidence is clear. Countries cannot afford to tolerate children being exposed to any unhealthy food marketing if they are to adequately serve the current and future interests of their citizens, and mandatory regulations can reduce this exposure in children. The newest evidence from natural experiments in Chile show how comprehensive mandatory regulation can reduce the exposure of children and adolescents to food marketing [16,17]. Chile first limited the promotion of products high in added sugar, sodium, saturated fat and/or energy in spaces where at least 20% of the audience were children and limited the use of child-directed content in the promotions. Later, the restriction was expanded to include all advertising of these products on television from 6 AM to 10 PM. Significant reductions in children’s exposure were seen at each stage, with a much greater decline when the daytime TV ban was instituted [16,17].

The new WHO guideline on food marketing policy marks a significant and positive step in protecting children, and future iterations must go even further to encourage the sea change needed for worldwide collaboration in tackling the role of food marketing in global health. Likely, a focus on HFSS products will not be enough to fully address nutrition-related NCDs. As the WHO’s new guideline was released, a larger concern has been developing about the link between consumption of ultra-processed foods and mortality [18]. Countries will need to adopt comprehensive definitions of marketing content, placement, and targeting strategies, and of unhealthy food and beverage products and brands including ultra-processed foods not covered by HFSS criteria, if they are to fully protect all children from unhealthy food marketing and its influence on their health trajectories.
